# Is Aboriginal Food Less Allergenic? Comparing IgE-Reactivity of Eggs from Modern and Ancient Chicken Breeds in a Cohort of Allergic Children

**DOI:** 10.1371/journal.pone.0019062

**Published:** 2011-04-28

**Authors:** Matthias Egger, Claudia Alessandri, Michael Wallner, Peter Briza, Danila Zennaro, Adriano Mari, Fatima Ferreira, Gabriele Gadermaier

**Affiliations:** 1 Christian Doppler Laboratory for Allergy Diagnosis and Therapy, University of Salzburg, Salzburg, Austria; 2 Center for Molecular Allergology, IDI-IRCCS, Rome, Italy; 3 Department of Molecular Biology, University of Salzburg, Salzburg, Austria; Centre de Recherche Public de la Santé (CRP-Santé), Luxembourg

## Abstract

**Background:**

Hen's egg allergy ranks among the most frequent primary food allergies in children. We aimed to investigate sensitization profiles of egg allergic patients and compare *in vitro* IgE reactivities of eggs from ancient chicken breeds (Araucana and Maran) with those from conventional laying hen hybrids.

**Methodology:**

Egg allergic children (n = 25) were subjected to skin prick test, double blind placebo controlled food challenge, and sensitization profiles to Gal d 1–5 were determined by allergen microarray. IgE binding and biological activity of eggs from different chicken breeds were investigated by immunoblot, ELISA, and mediator release assays.

**Principal Findings:**

We found that Gal d 1 and Gal d 2 are generally major egg allergens, whereas Gal d 3–5 displayed high sensitization prevalence only in patients reacting to both, egg white and yolk. It seems that the onset of egg allergy is mediated by egg white allergens expanding to yolk sensitization in later stages of disease. Of note, egg white/yolk weight ratios were reduced in eggs from Auraucana and Maran chicken. As determined in IgE immunoblots and mass analysis, eggs from ancient chicken breeds did not differ in their protein composition. Similar IgE-binding was observed for all egg white preparations, while an elevated allergenicity was detected in egg yolk from Araucana chicken.

**Conclusion/Significance:**

Our results on allergenicity and biological activity do not confirm the common assumption that aboriginal food might be less allergenic. Comprehensive diagnosis of egg allergy should distinguish between reactivity to hen's egg white and yolk fractions to avoid unnecessary dietary restrictions to improve life quality of the allergic child and its family.

## Introduction

Approximately 15% of the overall population suffers from IgE-mediated adverse reactions upon the ingestion of several kinds of food. According to the sensitization process, this disease can be divided into primary and secondary food allergies, affecting 9.4% and 5.5% of the population, respectively [1]. Adults often develop secondary food allergies as a consequence of primary sensitization through inhalant or contact allergens. Secondary food-induced symptoms are predominantly local and attributed to homologous molecules, which are recognized by cross-reactive IgE antibodies. In contrast, primary food allergy mainly affects children, is frequently characterized by severe symptoms, and the sensitization process, which takes place in the gastrointestinal tract, is mediated by the food allergen itself [2], [3].

Allergic reactions to hen's egg represent one of the most frequent primary food allergies affecting around 1.6% of children below the age of three, but are in the majority of cases outgrown before school age [2], [4]. So far, the official allergen list of the IUIS Allergen Nomenclature Subcommittee contains 6 chicken (*Gallus domesticus*) allergens while nine more IgE-binding egg proteins are listed in the Allergome database (www.allergome.org). Ovomucoid (Gal d 1), ovalbumin (Gal d 2), ovotransferrin (Gal d 3), and lysozyme (Gal d 4) constitute the most abundant proteins in the egg white fraction and have all been described as major allergens [5], [6], [7]. Gal d 1 is a highly glycosylated protein that is fairly resistant to heat and pepsin treatment and therefore, might cause severe allergic reactions upon consumption of cooked eggs [8], [9]. Gal d 2 constitutes 54% (w/w) of the total egg white proteins but seems less stable to heat and gastric digestion [7], [10]. Gal d 3 is an iron-binding heat-labile allergen that accounts for around 12% of the total egg white weight [11]. Sensitization to Gal d 4 can be exceedingly inconvenient, as it is commonly used as food preservative due to its antibacterial properties. Notably, egg white proteins are essential in the sweet cuisine constituting ingredients of many pastries and desserts [4], [5], [7]. Moreover, they have been revealed as occupational inhalant allergens in patients with baker's asthma [12], [13].

Although to a lower frequency, allergic reactions to yolk have been reported [11], [14]. Yolk represents an important emulsifier, and so far two allergens have been described at the molecular level. The first, alpha-livetin (Gal d 5), is partially heat-labile and belongs to the serum albumin protein family [6], [13]. Besides eliciting egg allergy in children, Gal d 5 has been implicated in the development of secondary egg allergy in adults sensitized to avian inhalant antigens, a phenomenon termed “bird-egg syndrome” [15], [16]. The second, vitellogenin (Gal d 6) has been identified recently and characterized as heat-resistant minor allergen in yolk [11].

Egg allergy has a significant effect on the quality of life and although symptoms are usually not life-threatening, severe anaphylactic reactions upon egg consumption have been reported [17]. As there is no efficient treatment for egg allergy so far, avoidance represents the only way to safely escape symptoms. However, as egg-derived components constitute ingredients of many cooked and manufactured food products, this approach is rather difficult and implies broad dietary restrictions [18]. The current tendency towards a deliberate, healthy, and balanced nutrition implicates a trend back to organic and natural foods. This heavily influenced food industry leading to customer-oriented changes in product development and marketing [19], [20], [21]. From the few hundred established chicken breeds only a small number is economically important as egg producers in poultry industry. Due to the worldwide demand, nowadays the majority of eggs is produced by high-performance laying hen hybrids [22]. Recently, eggs from ancient chicken breeds are getting promoted as aboriginal alternative trying to satisfy customer's requirements. Interestingly, the usage of laying hen hybrids in poultry industry since the 1950s is paralleled by a general increase in the prevalence of food allergy [23]. For this reason, we compared the allergenicity of eggs from conventional laying hen hybrids with those from ancient chicken breeds (Araucana and Maran). Within this study we investigated a cohort of 25 egg allergic children that were subjected to detailed *in vitro* and clinical allergy diagnosis.

## Results

### Egg allergic patients differ in their sensitization profiles

**Figure 1:**
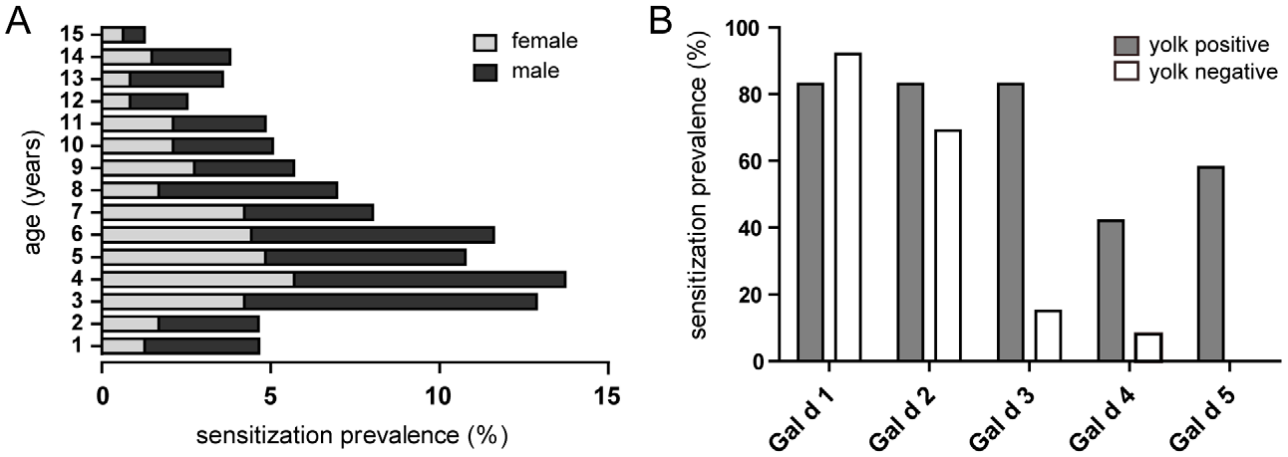
Age distribution and sensitization profile to hen's egg allergens. (A) Egg sensitized subjects (n = 474) were clustered according to age (1–15 years) and gender. (B) Sensitization prevalence to Gal d 1–5 of allergic patients reactive to egg white and yolk (P1–12) and those sensitized to egg white only (P13–25) are presented in grey (yolk positive) and white bars (yolk negative), respectively.

Studying a large cohort of 474 Italian subjects [24] displaying specific IgE to egg allergens revealed that egg sensitization predominantly affects male children (60%) in the age of 3 to 8 years (Figure 1A). For the present study we selected 25 patients displaying clinical symptoms upon egg consumption, which was confirmed by skin prick test (SPT) and double blind placebo controlled food challenge (DBPCFC) (Table 1). The average age of these children was 5.5 years and a stronger bias towards male gender was observed (20 male versus 5 female). The sensitization prevalence was highest for Gal d 1 (88%), Gal d 2 (76%), and Gal d 3 (48%), whereas reactivity to Gal d 4 and Gal d 5 were 24% and 28%, respectively (Table 1). As revealed by ELISA, 48% of egg allergic patients were sensitized to egg white and yolk (patients 1–12), whereas 52% displayed IgE antibodies with exclusive specificity for egg white components (patients 13–25). Notably, the concentration of egg white-specific IgE was significantly increased (*P* = 0.015) in the group of patients displaying additional sensitization to yolk (median 3.56 µg/ml versus 1.02 µg/ml). With 6.8±3.6 years (mean ± STD), the average age in this group was also slightly higher than for children belonging to the yolk-negative group (4.3±2.3 years). Moreover, the two groups differed in their allergen sensitization pattern (Figure 1B). In microarray experiments we found that Gal d 1 and Gal d 2 are relevant allergens in both patient groups, *i.e.* egg allergic children with and without yolk-specific IgE. By contrast, the sensitization prevalence to Gal d 3 and Gal d 4 was significantly higher in yolk-reactive patients (83% versus 15% and 42% versus 8%, respectively), even though those proteins are considered egg white allergens. As expected, IgE reactivity to Gal d 5 was exclusively detected in yolk-sensitized patients.

**Table 1:** Demographics and immunological characterization of hen's egg allergic patients.

Patient			SPT	ELISA				Symptoms recorded after DBPCFC	Reported Symptoms	ISAC				
#	gender	age		W	W^d^	Y	Y^d^			Gal d 1	Gal d 2	Gal d 3	Gal d 4	Gal d 5
**1**	m	10	19.3	6.92	4.54	5.57	5.61	AD, UR, AE	AD	5.28	5.58	4.65	7.31	10.18
**2**	m	11	43.1	1.20	1.40	1.39	1.48	AD, UR	AD	1.16	1.42	-	2.26	1.04
**3**	m	7	38.7	6.33	4.91	3.29	3.22	n.p.	GI, UR, AE, CO, RH, AS, AN	4.26	1.49	3.44	-	10.50
**4**	m	5	24.3	9.31	8.89	3.84	4.02	AD, GI	AD, GI	13.08	5.30	12.80	-	1.02
**5**	m	1	24.2	1.31	0.60	0.61	0.66	AD, UR	AD	2.81	2.73	6.48	4.09	-
**6**	m	7	22.4	1.98	1.61	0.33	0.53	n.p.	GI, UR, AE	26.50	9.38	5.34	1.82	-
**7**	m	3	17.3	1.40	-	0.60	0.51	AD, UR, AE	AD	-	1.35	3.93	-	0.64
**8**	m	9	22.4	4.76	4.84	1.88	2.10	AD, UR	AD	2.63	-	4.46	-	3.56
**9**	f	13	23.7	9.98	7.08	13.27	11.30	AD, UR	AD	2.73	-	11.69	-	21.28
**10**	m	4	24.1	2.35	2.72	0.67	0.73	AD, UR, AE	AD	2.40	4.02	1.14	-	-
**11**	m	7	21.5	7.36	5.00	3.16	3.46	GI	GI	2.47	1.68	1.25	-	-
**12**	m	4	15.8	0.63	0.71	0.45	0.39	UR	UR	-	1.62	-	0.83	-
**13**	f	7	14.9	0.91	0.77	-	-	AD, UR	AD	0.87	1.58	-	1.22	-
**14**	m	9	23.2	0.61	0.31	-	-	AD, GI	AD, GI	1.94	0.92	1.73	-	-
**15**	m	4	47.2	1.16	1.22	-	-	AD, UR	AD, UR	4.58	1.10	0.26	-	-
**16**	m	4	21.7	1.58	1.54	-	-	n.p.	GI, UR, AE, CO, AN	7.88	1.60	-	-	-
**17**	f	6	23.7	0.91	0.85	-	-	n.p.	GI, CO, RH, AS	2.50	1.36	-	-	-
**18**	m	4	26.7	1.02	1.35	-	-	AD, AE, UR	AD	1.11	1.54	-	-	-
**19**	m	1	45.7	4.64	3.21	-	-	AD, OAS, UR	AD, OAS	5.27	1.37	-	-	-
**20**	m	5	41.2	3.43	4.03	-	-	AD, GI, UR	AD, GI, UR	6.21	1.15	-	-	-
**21**	f	1	18.7	0.43	-	-	-	AD, AE	AD	-	0.13	-	-	-
**22**	m	3	8.1	0.45	0.26	-	-	n.p.	GI, UR	0.93	-	-	-	-
**23**	m	2	26.4	6.71	7.33	-	-	GI	GI	6.33	-	-	-	-
**24**	m	4	25.3	1.62	1.54	-	-	UR, AE	UR	5.55	-	-	-	-
**25**	f	6	27.3	0.76	0.74	-	-	AD, AE, UR	AD	3.07	-	-	-	-
**mean**	**f = 5 m = 20**	**5.5**	**25.9**	**3.11**	**2.62**	**1.40**	**1.36**	**AD = 16, UR = 15, AE = 7, GI = 5, OAS = 1**	**AD = 16, GI = 10, UR = 8, AE = 3, CO = 3, RH = 2, AS = 2, AN = 2,**	**4.38**	**1.81**	**2.38**	**0.79**	**2.01**

SPT were performed with commercially available egg white extracts and are presented as wheel area (mm^2^). Specific IgE antibodies were measured by ELISA with coated native egg white (W), denatured egg white (W^d^), native yolk (Y), and denatured yolk (Y^d^) extracts; results are expressed as specific IgE concentrations in µg/ml. IgE reactivity to *Gallus domesticus* allergens were determined by microarray using Immuno solid-phase allergen chips (ISAC) and given in as kU_A_/l. DBPCFC, double blind placebo controlled food challenge; AD, atopic dermatitis; AE, angioedema; AN, anaphylaxis; AS, asthma; CO, conjunctivitis; GI, gastrointestinal symptoms (including vomiting, diarrhea, and pain); n.p., not performed; OAS, oral allergy syndrome; RH, rhinitis; UR, generalized urticaria.

### Clinical manifestation of egg allergy

As illustrated in Table 1, allergic children predominately displayed skin-related symptoms (90%), while 35 and 25% were recorded with angioedema and gastrointestinal symptoms after food challenge. Forty percent of patients suffered from more than a single clinical manifestation and two of those experienced anaphylaxis. Patients with gastrointestinal symptoms, angioedema, and/or anaphylactic symptoms were usually sensitized to both Gal d 1 and Gal d 2. Apart from that, we did not observe any correlation between clinical symptoms and sensitization to distinct allergens. A single patient experienced local oral allergy syndrome, 4 patients displayed airway symptoms, and conjunctivitis was reported by 3 patients.

### Eggs of ancient chicken breeds display lower egg white to yolk weight ratios

Weights and pH values of eggs from Araucana, Maran, and laying hen hybrid chicken were determined in the laboratory (Table 2). No significant differences in the pH of egg white and yolk fractions could be observed among different eggs. Noteworthy, eggs from chicken breeds with increased laying performance produced heavier eggs, a circumstance that is desired by food industry and deliberately created by the breeder. Eggs from laying hen hybrids primarily feature comparatively enlarged egg white and reduced yolk fractions. Average weight ratios of egg white/yolk were significantly different (*P*<0.001) constituting 1.7 and 2.4 for ancient chicken breeds and modern laying hens, respectively.

**Table 2:** Characteristics of ancient and modern hybrid chicken breeds.

Property of breed	Araucana	Maran	Laying hen hybrid
Age	before 1500	late 1800s	since the 1950s
Origin	Chile	France	USA
Laying performance	∼180 per year	∼200 per year	∼320 per year
Egg shell color	green-white	dark brown	brown
Whole egg weight*	51.9±3.6 g	59.8±3.4 g	60.5±3.2 g
Egg white weight*	28.9±2.7 g	32.0±3.4 g	37.0±2.1 g
Yolk weight*	16.5±1.1 g	18.5±1.6 g	15.4±1.5 g
Ratio egg white/yolk (w/w)*	1.7±0.1	1.7±0.3	2.4±0.2
pH egg white	9.0	9.0	9.0
pH yolk	5.5	5.0	5.5

*Weights were determined and arithmetic means and standard deviations were calculated from 10 individual eggs.

### Eggs of different chicken breeds do not differ in their allergen composition

**Figure 2:**
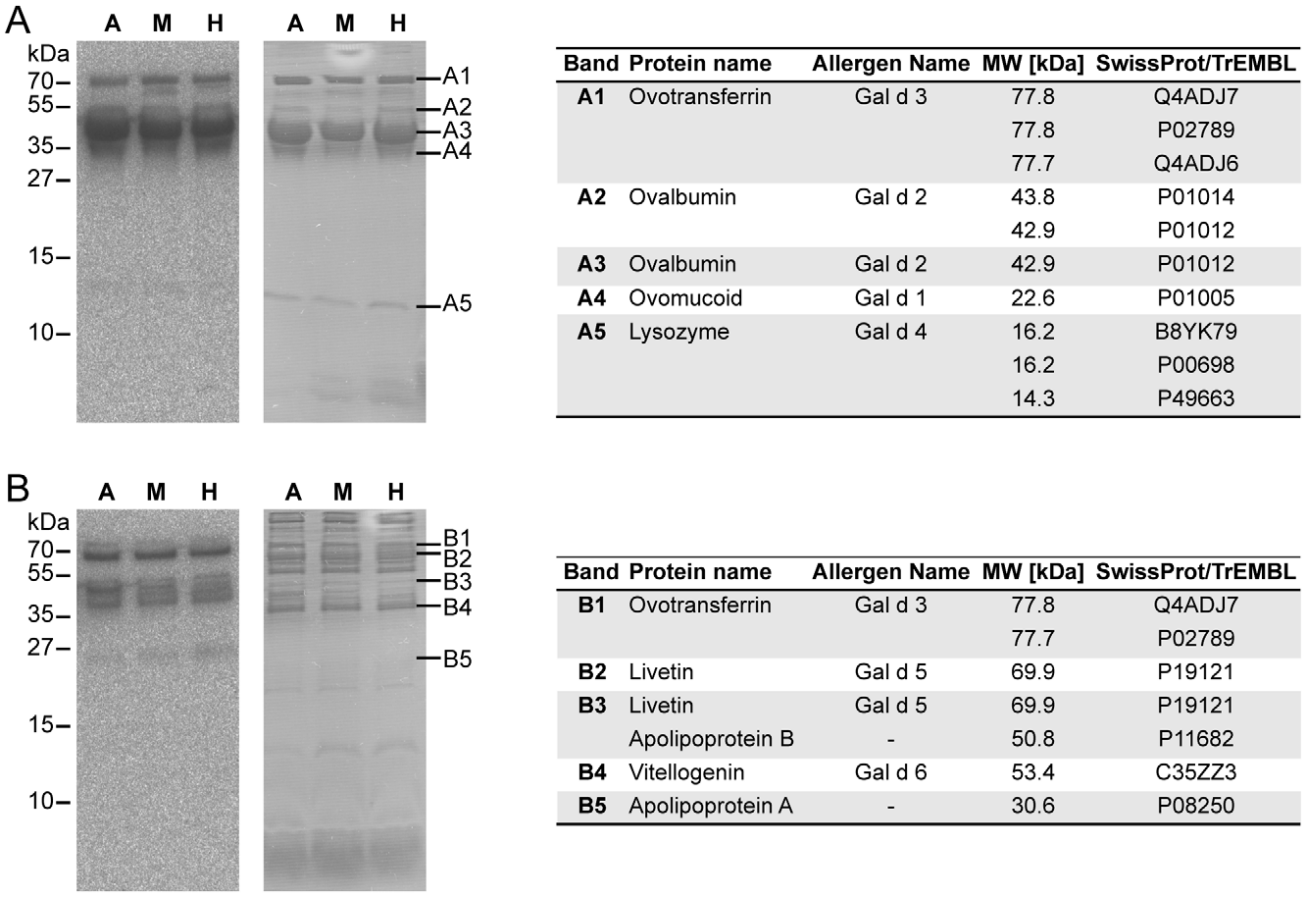
Identification of allergenic egg components. IgE-reactive egg white (A) and yolk (B) extract components from Araucana (A), Maran (M), and conventional laying hen hybrid (H) breeds were detected by IgE immunoblotting (left charts). IgE-reactive bands (A1–5 and Y1–5) were excised from polyacrylamide gels (right charts), and subjected to mass spectrometry analysis. Corresponding proteins, their molecular weights (MW), and SwissProt/TrEMBL accession numbers are given in the tables.

Egg white and yolk extracts from Araucana, Maran, and modern laying hen hybrids displayed similar protein composition and IgE-reactivity patterns as revealed by gel electrophoresis and IgE immunoblot analysis (Figure 2). Mass-spectrometry based identification of IgE-reactive bands demonstrated the presence of Gal d 1–4 in the egg white fractions from eggs of the three chicken breeds investigated. Noteworthy, we obtained only a single tryptic peptide derived from Gal d 1, presumably due to its enormous protein stability [25]. In the yolk fraction, Gal d 3, Gal d 5, and Gal d 6 were detected, and apart from the officially registered allergens, apolipoproteins were revealed as IgE-binding proteins.

### Thermal treatment of egg white and yolk has no significant influence on IgE reactivity

**Figure 3:**
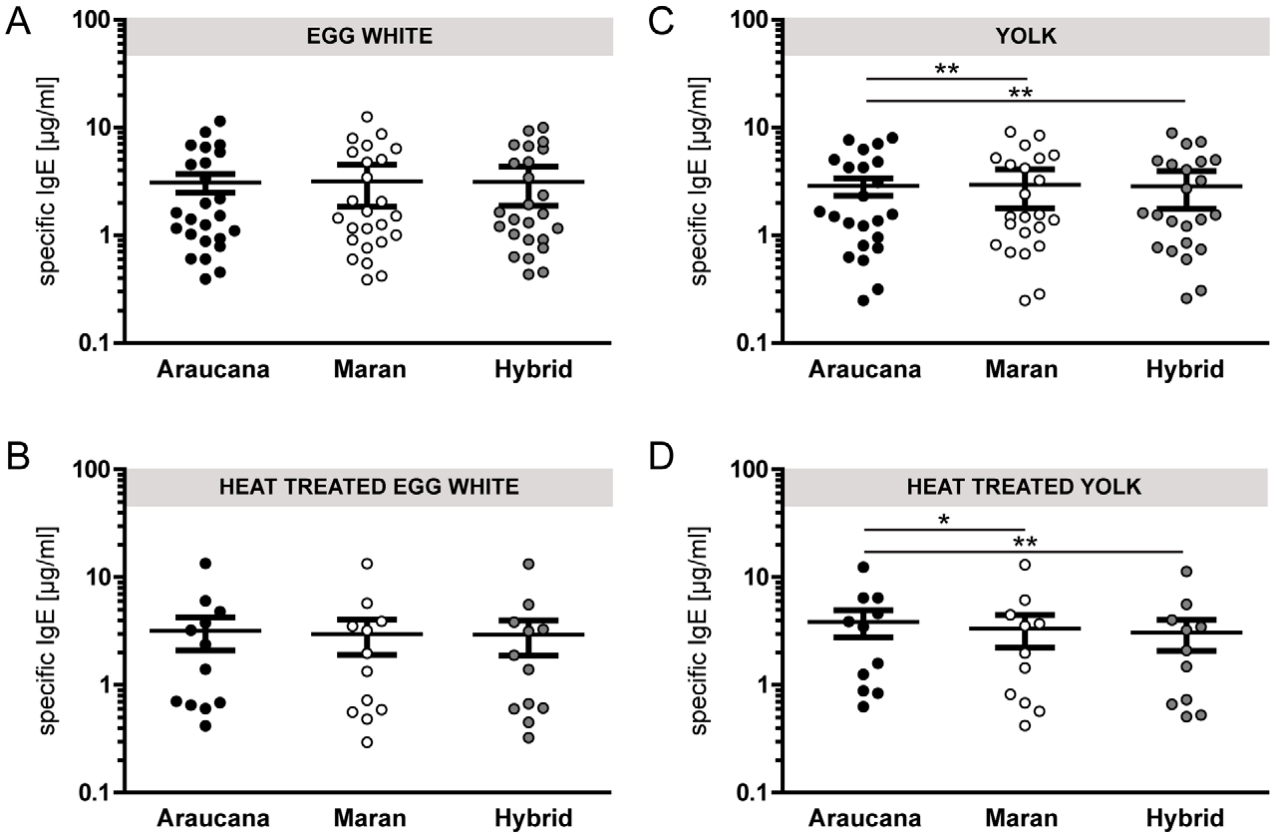
Allergenicity of egg white and yolk from different chicken breeds. Specific IgE levels to native and heat-treated egg white (A and B) and yolk (C and D) from Araucana (black circles), Maran (white circles), and conventional laying hen hybrid breeds (grey circles) were determined by ELISA. Circles represent values obtained from individual patients' sensitized to egg white (n = 25) and yolk (n = 12). Mean values are shown as solid line and whiskers indicate the standard error of the mean. Statistical analysis was performed using the Wilcoxon signed rank test. **<0.01; *<0.02.

ELISA experiments showed that thermal denaturation had only a minor impact on the IgE reactivity of egg extracts (Figure 3). No significant difference was observed between specific IgE concentrations to raw and heat treated egg white and to raw and thermally treated yolk of all eggs investigated. However, heat treatment abolished IgE reactivity to egg white extracts in 2 patients (patient 7 and 21), which were both negative for Gal d 1 (Table 1).

### Eggs of different chicken breeds display comparable IgE binding and biological activity

**Figure 4:**
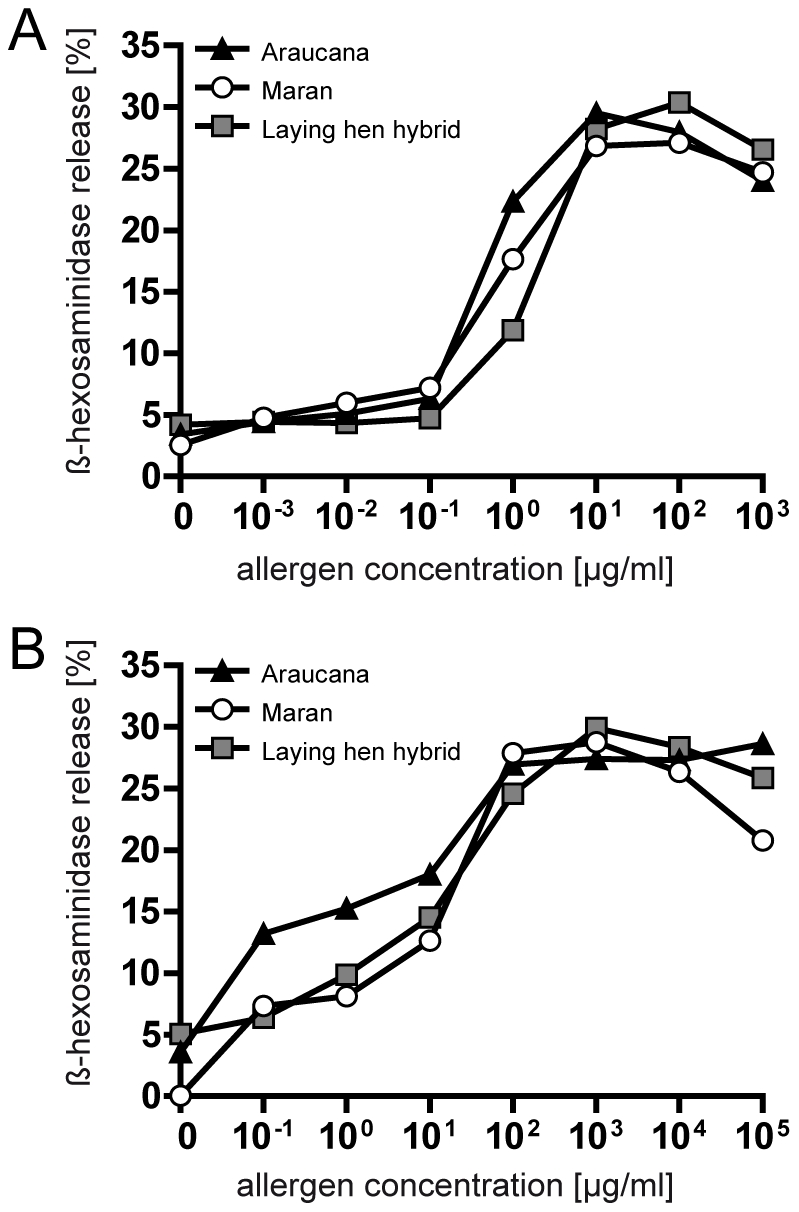
Biological activities of egg white and yolk from different chicken breeds. Transfected RBL-2H3 cells were passively sensitized with a serum pool of 8 patients. Mediator release was triggered with increasing concentrations of egg white (A) and yolk (B) extract.

ELISA experiments revealed similar levels of IgE reactivity of eggs from different breeds (Figure 3). Mean egg white-specific IgE titers were 3.09 µg/ml (Araucana), 3.17 µg/ml (Maran), and 3.11 µg/ml (laying hen hybrids); mean yolk-specific IgE titers 3.18 µg/ml (Araucana), 2.98 µg/ml (Maran), and 2.92 µg/ml (laying hen hybrids). Using the Wilcoxon signed rank test, we observed a difference for the IgE reactivity to egg yolk from Auraucana hen, which was evoked by 5/12 patients who demonstrated higher IgE values towards this breed. Similar IgE binding activities were reflected by a comparable mediator release capacity, with a slightly higher allergenic potential of egg yolk from Auraucana chicken (Figure 4). Highest degranulation values were observed at concentrations of 10 to 100 µg/ml for egg white and 100 to 10,000 µg/ml for yolk extracts. Thus, egg white seems to trigger a 100-fold stronger mediator release.

## Discussion

Although in the majority of cases outgrown before school age, egg allergy can drastically decrease life quality due to the omnipresence of egg containing food products. Avoidance is usually difficult and implies broad dietary restrictions [7], [17], [18]. However, strongly limited diets are frequently unnecessary and even unfavorable for the patient [26]. For proper patient management detailed diagnosis including molecule-based approaches [5], [27] and/or food challenges [28], [29], [30] may assist the clinician to provide dietary advice. It is well known, that temperature and food matrix are important aspects determining the allergenicity of food proteins [31]. In this context, our data suggest that half of the children in the present study are likely to tolerate yolk and yolk-containing foodstuff. Moreover, two (8%) of our patients were negative for heated egg white in ELISA and able to consume cooked egg white without problems (Alessandri *et al*, unpublished). On the molecular level, our and recent data [9] clearly indicate that the risk for adverse reactions upon ingestion of cooked eggs is linked to Gal d 1 sensitization. Noteworthy, a previous study showed that more than 50% of egg allergic patients tolerated ingestion of extensively heated egg and continued intake was even beneficial for the patient [32]. Our results evidence that IgE reactivity to Gal d 5 serves as marker for yolk sensitization. Since not all of the yolk sensitized patients reacted to this allergen, other molecules, *e.g.* the recently identified Gal d 6 [11] or apolipoproteins [33], could play a role in yolk allergy.

Although eggs from different birds contain cross-reactive allergens, the development of egg allergy seems to be restricted to distinct avian species [34], [35] and thus, some hen's egg allergic patients might tolerate consumption of eggs from duck or goose. Moreover, eggs from different chicken breeds might vary regarding their allergenicity. In fact, diverse apple cultivars have been demonstrated to differ in both, their allergen content and allergenic potential [36], [37]. Thus, aboriginal eggs from ancient chicken breeds might be favorable for the allergic patient. Accordingly, we compared the allergenic properties of eggs from conventional laying hen hybrids with those from Araucana and Maran ancient chicken breeds.

We did not find major dissimilarities regarding IgE reactivity or biological activity between eggs from modern and ancient chicken breeds. Noteworthy, egg yolk from Auraucana chicken even showed a slightly elevated allergenic potential. Based on these clear *in vitro* data showing no advantage of eggs from ancient breeds, food challenges with Auraucana and Maran eggs were considered of minor scientific benefit. Moreover, it has previously been shown that continuous egg intake boosts systemic IgE responses in allergic patients [38]. Hence, repeated food challenges would be ethically irresponsible as they bear unnecessary risks for the allergic children. Of note, differences in the nutritional composition of Araucana eggs, *i.e.* higher cholesterol and lower protein content, have been previously shown [39] and might have an impact on predisposed patients. Eggs from ancient breeds varied regarding their significantly decreased egg white/yolk weight ratio. Hence, for a minority of patients decreased exposure to egg white allergens and/or non-allergological factors that were out of scope of this study might deliver a possible explanation for better tolerability. Taken together, the fact that hen's egg allergy was rising in the last century might be explained by environmental factors rather than by consumption of eggs from novel high performance laying hen breeds that were not available in the past.

So which then came first, the chicken or the egg? From the allergological point of view this intriguing question might be answered as follows: Although representing a putative sensitizer in the “bird-egg syndrome” [14] and more rarely in children with meat allergy [35], [40], the chicken *per se* contributes little to primary egg allergy. By contrast, it seems that the egg, and in particular sensitization to egg white proteins, comes first. A picture is emerging in which the development of egg allergy seems to start with sensitization to Gal d 1 and Gal d 2. In the course of disease children raise additional IgE antibodies against other egg white (Gal d 3 and Gal d 4) and finally yolk allergens. This hypothesis is supported by our allergen profiling data and the fact that yolk-negative patients were by trend younger. Although additional yolk sensitization does not seem to cause more severe symptoms, it may worsen life quality because it enlarges dietary restrictions.

In summary, eggs from ancient chicken breeds cannot be considered less allergenic but might be better tolerated by some not severely affected patients due to their smaller egg white fraction. There are strong variations regarding the number and type of egg-containing foods that an individual patient can eat without experiencing adverse reactions. Therefore, detailed and molecule-based diagnosis distinguishing between sensitization to egg white and yolk is essential for improved patient management.

## Materials and Methods

### Ethics Statement

The study was approved by the Institutional Review Board of Istituto Dermopatico dell'Immacolata – IDI-IRCCS, Rome, Italy (n. 106-CE-2005), and signed informed consents were obtained from legal guardians.

### Patients and serology

Sera were obtained from the Center for Molecular Allergology at IDI-IRCCS in Rome, Italy. Egg allergic children were selected according to typical case history and positive skin reactivity. Skin prick tests (SPT) were performed as previously reported [41] and were recorded as wheal areas. Specific IgE levels to Gal d 1–5 were determined using the ISAC 89, VBC-Genomics, Vienna, Austria [42] according to previously reported protocols [24]. Diagnostic and clinical features have been recorded using an electronic allergy health record (InterAll, Allergy Data Laboratories s.c., Latina, Italy).

### Double-blind placebo controlled food challenge

Children were admitted to the Clinical Center in a fasting state. Administration of antihistamines, if used, was stopped at least 7 days before provocation test and none of the patients was under steroid treatment. Food challenge was carried out with commercially available raw eggs from hybrid hens in two different days. Eggs were mixed with 50 ml of non-sparkling mineral water plus two teaspoons of cocoa (Cacao Amaro, Perugina, Italy) and one spoon of white sugar (verum). After administration of an initial dose of 0.1 ml, amounts were increased to 1 ml and 10 ml in 30 minute intervals. Residual egg preparations were given until objective symptoms developed or until the equivalent of one egg was ingested.

### Egg characterization and sample preparation

Eggs from ancient chicken breeds, *i.e.* Araucana and Maran, were obtained from the Hölzl poultry hatchery in Moosburg, Germany. Medium-sized eggs from conventional laying hen hybrids were purchased at the local grocery. Average weights from whole egg, egg white, and yolk, as well as egg white to yolk weight ratios were determined for 10 individual eggs from each chicken breed. Moreover, pH values of egg white and yolk fractions were measured with Whatman pH indicator stripes. For preparations of allergen extracts, 3 ml samples of yolk and egg white fractions were taken from 10 individual cracked eggs. To avoid egg white contaminations, yolks were gently punctured with a syringe and samples were taken carefully. Pooled samples were extracted overnight at 4°C in PBS pH 7.4 containing 2% polyvinylpyrrolidone, 2 mM phenylmethanesulfonylfluoride, 2 mM benzamidine, and 2 mM ethylenediaminetetraacetic acid at a concentration of 1 g/ml. After centrifugation at 21,000 g and 4°C for 30 min, samples were sonicated, and filtered through a 0.45 µm filter unit (Millipore, Bedford, MA, USA). Protein concentrations were determined by the method of Bradford, and egg extracts were stored at 4°C.

### Gel electrophoresis and IgE immunoblotting

Egg white and yolk extracts were separated by SDS-PAGE under reducing conditions and stained with GelCode® Blue Reagent (Thermo Scientific, Waltham, MA). For immunoblots, extracts were separated by gel electrophoresis and electroblotted onto nitrocellulose Protan membranes (Schleicher & Schuell, Dassel, Germany). After blocking unspecific binding sites with 25 mM Tris HCl, pH 7.5, 0.5% bovine serum albumin (w/v), 150 mM NaCl, and 0.05% Tween 20 (v/v), membranes were incubated with a serum pool (n = 25) diluted 1∶10 in the blocking buffer. Bound IgE was detected using ^125^I-labelled anti human IgE (BSM Diagnostika, Vienna, Austria). Radiographic signals were visualized by overnight exposure to imaging plates and subsequent evaluation using a BAS-1800II Phosphor Imager scanner (Fuji Film, Tokyo, Japan).

### Mass-spectrometry based allergen identification

IgE-reactive bands were excised from polyacrylamide gels and subjected to in gel digestion using the ProteoExtract All-In-One Trypsin Digestion Kit (Calbiochem, Gibbston, NJ). Tryptic peptides were separated by capillary reversed phase high pressure liquid chromatography (rpHPLC) directly coupled to a Quadrupole-Time of Flight mass spectrometer (QTof Ultima Global, Waters, Milford, MA) equipped with a Nanoflow spray head. Obtained mass data were processed and analyzed using the software Protein Lynx Global Server version 2.2.5 (Waters, Milford, MA) and evaluated using the SwissProt/TrEMBL database.

### Enzyme-linked immunosorbent assay

Maxisorp plates (Nalge Nunc, Rochester, NY) were coated with 250 ng of egg extracts in PBS pH 7.4 overnight at 4°C. To assess IgE reactivity to heat-denatured egg allergens, egg extracts were incubated for 15 minutes at 95°C before use. After blocking unspecific binding sites with Tris-buffered saline pH 7.4, 1% bovine serum albumin (w/v), and 0.05% Tween 20 (v/v), coated antigen was incubated with patient's sera overnight at 4°C. Bound IgE was detected with an alkaline phosphatase-conjugated monoclonal anti human IgE antibody (BD Biosciences, Franklin Lakes, NJ). Specific serum IgE levels were calculated based on a semi-quantitative ELISA using purified human IgE (Serotec, Raleigh, NC) as internal standard.

### Mediator release assays

Rat basophil degranulation assays were performed as previously described [43]. Briefly, rat basophil leukemia (RBL)-2H3 cells transfected with the human high-affinity IgE receptor were passively sensitized with a serum pool from egg allergic patients (n = 8) reacting to both egg white and yolk. Basophil degranulation was triggered by addition of serial protein dilutions, and β-hexosaminidase release was measured by enzymatic cleavage of the fluorogenic substrate 4-methyl umbelliferyl-N-acteyl-β-glucosaminide (Sigma, St. Louis, MO). Results are expressed as percentage of total enzyme content of Triton X100-treated cells.

### Statistical analyses

Statistical evaluation of paired samples was performed using the Wilcoxon signed rank test; Mann-Whitney rank sum test and *t*-test were used for non-paired samples. A value of *P*<0.02 was considered statistically significant.
